# Reversal of Resistance in Targeted Therapy of Metastatic Melanoma: Lessons Learned from Vemurafenib (BRAF^V600E^-Specific Inhibitor)

**DOI:** 10.3390/cancers10060157

**Published:** 2018-05-24

**Authors:** Antoni Xavier Torres-Collado, Jeffrey Knott, Ali R. Jazirehi

**Affiliations:** Department of Surgery, Division of Surgical Oncology, and the Jonsson Comprehensive Cancer Center, David Geffen School of Medicine at UCLA, University of California at Los Angeles, Los Angeles, CA 90095, USA; axtorres@gmail.com (A.X.T.-C.); Jeffknott04@gmail.com (J.K.)

**Keywords:** apoptosis, targeted therapy, vemurafenib, melanoma, resistance, signal transduction, sensitization

## Abstract

Malignant melanoma is the most aggressive form of skin cancer and has a very low survival rate. Over 50% of melanomas harbor various BRAF mutations with the most common being the V600E. BRAF^V600E^ mutation that causes constitutive activation of the MAPK pathway leading to drug-, immune-resistance, apoptosis evasion, proliferation, survival, and metastasis of melanomas. The ATP competitive BRAF^V600E^ selective inhibitor, vemurafenib, has shown dramatic success in clinical trials; promoting tumor regression and an increase in overall survival of patients with metastatic melanoma. Regrettably, vemurafenib-resistance develops over an average of six months, which renders melanomas resistant to other therapeutic strategies. Elucidation of the underlying mechanism(s) of acquisition of vemurafenib-resistance and design of novel approaches to override resistance is the subject of intense clinical and basic research. In this review, we summarize recent developments in therapeutic approaches and clinical investigations on melanomas with BRAF^V600E^ mutation to establish a new platform for the treatment of melanoma.

## 1. Metastatic Melanoma: Basics and Treatment Options

Melanoma has a 10–15% five-year survival rate and is considered as the most aggressive form of skin cancer. It accounts for over 80% of skin cancer mortalities and is the 6th most prevalent cancer in the US [1,2,3,4,5]. Various treatments are available but do not always extend survival and are usually associated with toxicity. These modalities include surgery, radiation therapy, chemotherapy, and immunotherapy [6]. Excision of a tumor is advantageous for those people who have a higher chance of survival from surgery than from any other treatment. These individuals include those with fully resectable melanoma and not with disseminated disease. Patients with solitary subcutaneous metastasis could benefit from surgical removal of the tumors rather than from any other form of treatment [7]. Patients with sole lesions vs. those with multiple lesions present a five-year survival rate of 12% and 0%, respectively. However, irrespective of the extent of the excision, patients are still unlikely to achieve long-term survival rates [6].

Radiation therapy is used infrequently for cutaneous melanoma. Previously, melanoma was viewed as radio-resistant, but more recent clinical studies have invalidated that disposition [8,9]. Radiation therapy is utilized in patients to destroy and shrink tumors by induction of DNA damage to prevent their growth [8].

Cytotoxic chemotherapy involves administration of antimicrotubular agents, dacarbazine, temozolomide, and nitrosoureas. However, upon treatment some side effects including fatigue, alopecia, and hypersensitivity reactions can develop [10]. Dacarbazine, temozolomide and nitrosoureas are all alkylating agents. Dacarbazine is especially popular for the treatment of melanoma and has a response rate of 16% with an overall survival of eight months [11,12]. Dacarbazine was also compared to vemurafenib (BRAF^V600E^) in Phase III clinical trials [13].

Immunotherapy regimens include interferon (IFN)-alpha 2β, Interleukin-2, cytotoxic T-lymphocyte antigen-4 (CTLA-4) blocking antibodies, dendritic cell vaccines, and tumor infiltrating lymphocytes (TIL) adoptive T-cell therapy [5,10,14,15]. IFN-alpha 2β induces apoptosis in melanoma lines via a caspase-dependent manner [16]. It was the first agent with significant survival benefits in clinical trials with a survival rate of 3.82 years [17]. IFN-alpha 2β treatment causes adverse side effects such as fever, psycho-cognitive impairment, and fatigue, which is limited to stage IV melanoma patients [18]. Interleukin-2 (IL-2) is also utilized in the treatment of metastatic melanoma, which received FDA approval in 1998. This cytokine triggers the immune system by stimulating T-cell and natural killer (NK) cell proliferation and function. Some patients responded very well to treatment with a response rate of 16% in an analysis of 270 patients treated with IL-2, and none of these patients experienced any disease progression after five years [10,19]. However, IL-2 also has considerable side effects such as hypotension, cardiac arrhythmias, delirium, and rash [10] that requires hospitalization and this therapy did not improve overall survival [20,21].

Antitumor immune responses are vital in the treatment of melanoma. CTLA-4 blocking antibodies (ipilimumab and tremelimumab), bind to the CTLA-4 antigen and increase immune response against tumor cells. CTLA-4 antigen is a homologue of CD28, is expressed on activated T-lymphocytes and acts as an adverse regulator of T-cell activation. Ipilimumab and tremelimumab have similar response rates of about 7–10%, however, the response is delayed and takes up to 12 weeks to develop [22]. Ipilimumab has been administered in Phase III trials, where monotherapy improved overall survival rate of the melanoma patients [23]. The toxicities include immune-related enterocolitis, hepatitis, and dermatitis [24]. Vaccines with dendritic cells (DCs) might also be a beneficial treatment. DCs are antigen presenting cells that activate naïve T-cells directing adaptive immunity. However, DCs are not antigen specific [15]. DC-vaccinated melanoma patients show a pronounced T-cell proliferation. Hypersensitivity develops only when the patients are injected with mature DCs that induces immune response [25]. Treatment with the vaccine does not seem to have adverse side effects [26]. Thus, DC vaccines could be an effective form of immunotherapy battling against disseminated melanoma. Adoptive T-cell therapy (ACT) isolates, expands, and infuses tumor-infiltrating lymphocytes (TIL) in patients [27,28]. These lymphocytes are transduced with high affinity T cell receptors against specific tumor antigens with a clinical response rate over 50%, which reveals it as a successful treatment against metastatic melanoma [29]. TIL therapy is effectively induced in more than 60% of all patients and has higher response rates than ipilimimab or IL-2 [14,30].

## 2. Targeted Therapy in the Treatment of Metastatic Melanoma

Mitogen activated protein kinase (MAPK) pathway controls cellular proliferation, differentiation, and survival. Extracellular signal-regulated kinase (ERK) pathway is one of the major MAPK signaling pathways and is primarily involved in the proliferation and survival of cancer cells, leading to tumor growth [31]. A GTPase RAS once actively GTP-bound, activates RAF (ARAF, BRAF or CRAF) by recruiting the RAF proteins to the plasma membrane. BRAF is most easily activated by RAS and once mutated, signals as a monomer independent of upstream stimuli [32,33,34,35]. Receptor tyrosine kinases usually activate this signaling cascade of the pathway [36,37,38]. Various BRAF mutations occur in 60% of patients with melanoma, in which 90% of these mutations is the glutamic acid base substitution for valine at codon 600 (BRAF^V600E^). Mutations lead to constitutive activation of MAPK pathway, which causes cancer cell proliferation and a 500-fold increase in activity compared to wild type protein [39,40]. Vemurafenib (PLX4032, RG7204), an oral serine-threonine kinase inhibitor, is BRA^FV600E^ specific inhibitor, which has improved survival rate in melanoma patients. This drug preferentially inhibits the MAPK pathway and inhibits phosphorylation of MEK and ERK, which induces cell cycle arrest and triggers apoptosis in cells solely harboring the BRAF^V600E^ mutation [13]. The drug was discovered via the scaffold-based approach. This strategy is utilized for the identification of inhibitors of cyclic nucleotide phosphodiesterases. The tail of the inhibitor was found to bind to a pocket from ATP ribose triphosphate tail. Cell lines that bare the BRAF^V600E^ mutation are more sensitive to the BRAF inhibitor relative to the cells that do not possess the mutation [41]. The specificity of vemurafenib was initially tested via western blots to show a dose and V600E dependent inhibition of phosphorylated ERK. Unlike in the wild types cells (WT), dose increase of vemurafenib inhibited RAF signaling and increased growth arrest in the mutant cells. Prolonged exposure to vemurafenib does not decrease its selectivity for the V600E mutation. The inhibitory concentration of vemurafenib was shown to be 31 nmol/L at 50% (IC50) [39]. Owing to the successful in vitro and in vivo testing, vemurafenib exhibits as a promising drug against melanoma with cells expressing a BRAF^V600E^ mutation and has 10-fold greater selectivity for the mutation relative to the wild type cells [39,41]. But the eventual relapse and bypass of the melanoma cells treated with vemurafenib is due to the resistance mechanisms that progress over time. Another MEK inhibitor, AZD6244, also efficiently inhibits MAPK pathway. Nevertheless, their benefits are often offset since they impair T-lymphocyte function [42]. However, the BRAF^V600E^ inhibitor GSK2118436 targets cells with the mutation and was combined with ACT with the intent to alter the function of T-cells. In contrast to MEK inhibitors, the drug proved not to suppress patient lymphocytes. Another MEK inhibitor, PD0325901, in Phase I trials produced significant decrease in phosphorylation of ERK and disease stabilization [43,44]. Induction of IL-1, a tumor and fibroblast cytokine, promotes stromal cell-mediated immunosuppression in cells with a BRAF^V600E^ mutation. IL-1 is important in tumor progression [45]. Mutant BRAF can prompt T-cell suppression. BRAF^V600E^ induced IL-1 exacerbates immune suppression via stimulation of tumor associated fibroblasts (TAF) by melanomas. Due to TAF activity, tumor cells evade natural killer (NK) cells allowing tumor progression. Since IL-1 regulates immune suppression, it could exert inhibition on melanoma antigen-specific cytotoxic CD8+ T-lymphocytes. Vemurafenib is thought to be able to impede IL-1 production in melanoma due to BRAF^V600E^ inhibition. When BRAF^V600E^ is repressed, melanomas are relieved of TAF activity to instigate CTL activity [46]. Therefore, targeted blockage of IL-1 might be beneficial to patients suffering from BRAF^V600E^. Dabrafernib (GSK2118436) is another selective ATP competitive BRAF^V600E^ inhibitor. Just like vemurafenib, the inhibitor has selectivity towards the mutant BRAF and not the wild type [47]. The clinical trials included patients with V600E, V600K, V600D mutations and patients with untreated brain metastasis showing a progression free survival rate of 8.3 months. The drug scored similar results as vemurafenib in dose-escalating studies. Response rate in melanoma patients harboring V600E/K mutations was about 60% in Phase I and 59% in phase II [48]. The drug shows promise in combating metastatic melanoma. RAF265 is a BRAF^V600E^ inhibitor that induces tumor regression and decrease in growth. The responses were 16% in patients with a BRAF mutation and 13% in patients with WT BRAF. The results are moderate relative to other BRAF inhibitors [49]. The drug might cause more toxicity rather than enduring any feasible antitumor activity.

The Bcl-2 gene family includes antiapoptotic and proapoptotic members that are employed in targeted cancer therapy. Down regulation of antiapoptotic Bcl-2 can be achieved by oblimersen sodium oligonucleotide. As a result, melanoma tumor cells are rendered sensitive to apoptosis. Responses longer than six months were attained in metastatic melanoma patients treated with oblimersen sodium oligonucleotide [50]. Clinical investigation was implemented to investigate possible therapeutic use for tumors with RAS mutations, especially NRAS relative to KRAS and HRAS. It triggers MAPK and AKT pathways, drugs that counter this effect, such as tipifarnib or R115777, a farnesyltransferase inhibitor, were developed that impairs posttranslational modifications of RAS, inhibiting the MAPK pathway [51,52].

Novel drugs are required to inhibit constitutive activation of survival pathways. For instance, PI3K/AKT pathway regulates a cascade of signals that regulate cell proliferation, survival, and growth in melanomas as well as other cancers. PI3K related kinases (PIKKs) are homologs of PI3Ks [53]. Rapamycin analog CCI-779, showed anti-melanoma activity in preclinical models. The average overall survival in phase II trials was five months, and disease progression was ten weeks in patients treated with the analog and phenhydramine premedication. Yet, the agent itself cannot be used alone due to its weak activity [54]. ERK activation plays a role in angiogenesis by promoting vascular development via VEGF secretion. Mutations in ERK cause circumvention of immune response, senescence, tissue invasion, and metastasis [55]. Bevacizumab, an anti-VEGF monoclonal antibody, blocks the VEGF-induced angiogenesis [56]. Thalidomide has also been utilized in cancer therapy and is thought to block VEGF as well [57]. HSP90 (a chaperone protein for RAF kinases) inhibitor ganetespib is a promising novel drug that could be administered to melanoma patients with a BRAF mutation. The drug has potent anti-tumor activity in melanomas harboring BRAF mutations by inactivating MEK and ERK also causing depletion of CRAF, which when activated causes resistance to BRAF inhibitors. Genetespib was also combined with PI3K/mTOR inhibitors. Deactivation of MAPKK by ganetespib and activation of ERK by PI3K/mTOR inhibitors rendered melanoma cells to increased cell death [58]. HSP90 inhibitor XL888 also targets BRAF mutation inducing apoptosis in melanoma cell lines by decreasing PDGFRβ, COT, IGFR1, CRAF, ARAF, cyclin D1, AKT expression, increasing BIM expression, and downregulating Mcl-1, which are all part of resistance mechanisms (summarized below) [59].

## 3. Data on Clinical Treatment of Melanoma Patients with Vemurafenib

Due to its promising preclinical data, vemurafenib was further tested on patients with BRAF^V600E^. After successful phase I [60] and II trials [61], phase III trial (BRIM-3) compared vemurafenib and dacarbazine in patients who were previously untreated for metastatic melanoma and had a BRAF^V600E^ mutation. Vemurafenib and dacarbazine response rates were 48% and 5%, respectively. Vemurafenib group had a reduction of 63% in the risk of death and 74% in the risk of death or disease progression, relative to dacarbazine. Survival response for vemurafenib was 16 months compared to less than 10 months for dacarbazine. Side effects included rash, fatigue, photosensitivity, alopecia, arthralgia, nausea, and cutaneous squamous-cell carcinomas [13]. Due to its benefits, the FDA approved vemurafenib on 17 August 2011 [62]. Considering all phase studies, vemurafenib is a drug with augmented rates of overall survival in melanoma patients with a BRAF^V600E^ mutation.

## 4. Side Effects Associated with Vemurafenib Treatment in Melanoma Patients

Even though vemurafenib proved to be beneficial to patients, toxicity still applies. Some side effects included fatigue, diarrhea, nausea, arthralgia, photosensitivity, rash pruritus, and alopecia. Palmoplantar dysesthesia and keratosis pilaris occur in 1/3 of the patients [13,60]. Squamous cell carcinoma and keratoacanthoma were seen in 18% of patients treated with vemurafenib [13]. Cutaneous side effects arise due to MAPK pathway activation via vemurafenib therapy that lead to growth of lesions. Su et al. investigated the role of RAS mutation and squamous cell carcinoma. The results revealed that RAS mutations are frequent in this disease after vemurafenib treatments [63]. Paradoxical activation of MAPK pathway effect on BRAF wild type cells is another detrimental side effect of vemurafenib. In BRAF wild type melanoma cells, the drug stimulated downstream signaling, which increased tumor cell mobility and proliferation in cells with NRAS Q61L mutation. Treatment with vemurafenib is exclusively beneficial to BRAF^V600E^ mutant cells and harms the wild type cells causing detachment of cells and initiation of growth [64].

One study investigated vemurafenib and neutrophilic panniculitis. Two cases of this adverse side effect have shown that none of the patients had an immunity issue or an infection that might have contributed to the neutrophilic panniculitis but were treated with vemurafenib. One patient showed recovery while the other had recurrences. Therefore, the drug could have contributed to the toxicity [65].

## 5. Postulated Resistance Mechanisms to Vemurafenib Treatment in Melanoma

Melanoma cells seem to be able to adapt to selective pressures. Progression free survival in melanoma in patients treated with BRAF inhibitor vemurafenib is limited due to the developed resistance over an average of 6–8-month period [66]. Various mechanisms are thought to play a role causing eventual resistance to the drug. The vital mechanisms involved are the paradoxical hyperactivation of MAPK pathway, reactivation of mitogen-activated protein kinase 8 (COT), loss of phosphatase and tensin homolog (PTEN), PI3K/AKT/mTOR amplification, CRAF dimerization, suppression of BIM expression, increased cyclin D1, upregulation of N-RAS mutations, and high expression levels of platelet derived growth factor beta (PDGFRβ) and epidermal growth factor receptor (EGFR) [67,68,69,70,71,72,73,74]. Regulation of transcriptional events and phosphorylation through MAPK pathway causes cells to proliferate, avoid apoptosis, migrate, and invade [35].

COT is MAPK pathway agonist with kinase activity that causes resistance to the BRAF inhibitor through activation of MEK/ERK pathway by phosphorylation. Direct mutation of this kinase may also cause a reactivation of the pathway, however, RAF signaling is not essential for ERK activation by COT. Johanessen et al. demonstrated that when cells are treated with a BRAF inhibitor, overexpression of WT COT results in constitutive phosphorylation of ERK and MEK. Ectopic COT expression in melanoma cells lines cause a decreased sensitivity not only the BRAF inhibitor, but also to MEK inhibitors CI-1040 and AZD6244. Single agent therapy has an imminent relapse due to eventual resistance to treatment [72].

Receptor tyrosine kinases (RTKs) that are upstream of P13K/AKT, such as IGF-IR and PDGFR-B, have phosphorylating activity, which might contribute to the resistance of BRAF inhibitors [67,75,76]. For instance, RTKs are overexpressed in vemurafenib resistant melanoma cell lines. RTKs, such as PDGFR-B and EGFR, contain extracellular ligand recognition and cytoplasmic tyrosine kinase domains that transmit information through phosphorylation and are essential for cell growth [77]. PDGFRβ displayed elevated activation-associated tyrosine phosphorylation and were positive for a melanoma marker, melanoma antigen recognized by T-cells 1 (MART1). The activation of the receptor results in the activation of ERK pathway. G0/G1 cell cycle arrest occurs when PDGFRB is knocked down by shRNAs [74]. Treatment of mutant cells with RTK inhibitor, gefitinib, inhibited growth of melanoma cells and decreased ERK phosphorylation [78]. Resistance cells also express higher surface levels of insulin growth factor receptor 1 (IGF-1R). Even though IGF-1R promotes the activation of P13K, as will be discussed later, it has no consequence on the MAPK pathway [79].

Deletion or functional loss of a tumor suppressor and a negative regulator of P13K/AKT pathway PTEN, occurs in 5–20% of melanomas. Upregulation or mutation in AKT allows the P13K/AKT/mTOR signal transduction pathway amplification. PTEN is usually blocked by NRAS oncogenic mutations, silencing mutations or AKT amplification. This further confers resistance to vemurafenib and increases cell survival and apoptosis. Resistant cell lines have increased AKT3 signaling when exposed to PLX4032. AKT3 contains a point mutation E17K, which activates AKT pathway influencing mTOR activation.

RAS isoforms NRAS, KRAS, and HRAS are small GTPases that regulate cell proliferation and growth [80]. RAS can also upregulate IL-6 secretion that can promote cell growth on cancer cells. NRAS activating mutations are detected in 15% of melanomas, but KRAS and HRAS mutations are rare [81,82]. The most common mutations are at codons 12 and 61, which leads to abnormal regulation of RAS and accumulation of RAF-GTP [83]. Initiation of growth phase of melanoma cells is thought to be associated with RAS mutation [84]. Some studies have shown vemurafenib to increase proliferation of growth factor dependent NRASQ61L mutant in melanoma cells and increased mobility of tumor cells [64]. It was assumed that a resistant mechanism, which involved NRAS, was its secondary mutation. However, Nazarian et al. investigated the predisposition and demonstrated that even though NRAS is upregulated in vemurafenib resistant cells lines, it is not due to the NRAS secondary mutation that would prevent the drug from binding to BRAF^V600E^ [74].

MAPK signaling is hyperactivated by mutated NRAS that can activate CRAF, a RAF isomer, and BRAF heterodimerization and pathway switching, which can bypass vemurafenib sensitivity of cancer cells [85]. When BRAF mutants are impaired, CRAF activity is stimulated by BRAF through phosphorylation of CRAF, and MEK is activated signaling to ERK [44]. Within the BRAF/CRAF or CRAF/CRAF dimers, CRAF is activated by the dimmer interaction that is bound by the RAF inhibitor, following by recruitment of CRAF to the plasma membrane [64,85,86,87]. It is also due to CRAF activation that vemurafenib paradoxically induces the MEK/ERK pathway in BRAF WT cells even though inhibiting MEK/ERK phosphorylation in BRAF tumors with a BRAF^V600E^ mutation [30].

Dimerization could also be influenced by a BRAF^V600E^ abnormal splicing. Some cells that are resistant to vemurafenib express a variant form of BRAF^V600E^, of 61 kDa, which has a deletion in exon 4 and 8. This variant, p61BRAF^V600E^, exhibits dimerization in cells with low RAS activation relative the non-spliced BRAF^V600E^, since exons 4 and 8 are in the RAS binding domain [85]. This binding domain is necessary for RAF activation. The aberrant splicing is believed to be a result of a mutation and loss of function [39]. A mutation that eradicates the p61BRAF^V600E^ dimerization counters the effect of resistance and the cells become sensitive to the BRAF inhibitor again, which will subsequently initiate cell death [32].

Expression of Cyclin D1, an oncogene that allows cells to enter the cell cycle and regulates cyclin dependent kinases (CDK), may also contribute to BRAF inhibitor resistance. Cyclin D1 regulates the activity of CDK4 through binding and down-regulation of CDK inhibitor, p27, and demonstrates increased levels of cancer cell proliferation [69,88,89]. RAS dependent transformation also requires activation of cyclin D1 and downregulation of p27 [90]. In addition, senescence of melanocytes is bound with downregulation of CDK4 [91]. BRAF^V600E^ causes proliferation of melanoma cells by overcoming G1 phase constraint influencing cyclin D1 production in mid-G1 phase, which induces CDK4 activity [69].

RAF/MEK/ERK pathway is also inhibited via Bim, a proapoptotic member of the Bcl-2 family that binds with high affinity to antiapoptotic Bcl-2 proteins Bcl-2 (overexpressed in 80% of melanomas), Bcl-w, Bcl-XL, and Mcl-1 [92]. It has been shown that the BRAF inhibitor treatment enhances levels of Mcl-1, which is a pro-survival protein that prevents apoptosis in cancer cells [81]. ERK pathway inhibits apoptosis by phosphorylating Bad and Bim [93,94]. Bim inhibits the RAF/MEK/ERK pathway in melanoma, which causes tumor cell death. When Bim is phosphorylated, it is proteasomally degraded and it no longer associates with Bax, a proapoptotic factor. Bad’s phosphorylation disrupts the interaction with antiapoptotic Bcl-2, which allows to the cancer cells to survive [94]. Differences in Bim and Bad expression may allow some mutant cells lines to stop proliferating but not die from vemurafenib [95]. Jiang et al. also investigated the isoform of Bim(S) that is shown to be the most potent inducing isoform of Bim relative to Bim(EL) and Bim(L). BRAF^V600E^ inhibition triggers Bim(S) splicing by splicing factor SRp55, and when the mutation is expressed apoptosis and induction of Bim(S) decreases [96]. Expression of Bim is determined by PTEN activation. Thus, deregulation of PTEN reduces Bim binding and increases its suppression, which in turn will inhibit the ERK pathway [59].

Protein c-KIT is also known to contribute to the vemurafenib resistance of melanoma cells. The protein is a growth factor receptor in epidermal melanocytes. Its roles include migration and differentiation of melanocytes [97]. Abnormalities in c-KIT include mutations and amplification of this gene. Imatinib inhibits the tyrosine-kinase activity of c-KIT and has been shown to induce tumor regression in patients [98,99]. Clinical research conducted experiments on patients with melanomas from mucosa, acral skin, and skin with and without chronic sun-induced damage. All the patients demonstrated genetic abnormalities affecting KIT that frequently appeared on these various types of melanomas. Increased KIT signaling in melanoma could be a result of mutation. Cells that have mutations in KIT usually have a specific growth pattern where they are lined up in lentiginous growth as single cells in progression stage that is followed by invasion [100]. However, other findings affirm that KIT is downregulated in melanoma progression [101,102].

Reactivation of MAPK pathway due to resistance to vemurafenib could also be caused by fibroblast growth factor receptor 3 (FGFR3). The enhancement of the receptor is linked to RAS and MAPK activation. Inhibition of the receptor confers cancer cell sensitivity. FGFR3/RAS signaling pathway is another mechanism of resistance that could deem favorable as a targeted therapy. Activation of FGFR3 controls RAS activation in BRAF^V600E^ melanoma cell lines and reduces the sensitivity to BRAF inhibition, which results in cancer cell proliferation, growth, and survival. Levels of p-FGFR3 are increased in cells that are vemurafenib resistant in BRAF^V600E^ mutation specifically due to activation of downstream ERK. However, no phospho-AKT is detected in the melanoma cell lines conveying that the receptor does not take much part in the AKT pathway [103].

In the last years new mechanisms have been described to be implicated in the acquisition of Vemurafenib resistance in melanoma. One factor recently reported to be related to molecular defeat of BRAF inhibition is P21-activated kinase 1. Interestingly this kinase has been observed to be over-expressed in several tumor types [104]. Another mechanism recently uncovered involves ID3, a molecule implicated in cell migration and expression of SOX10 and MITF. This gene was found to be significantly unregulated (*p* = 0.00077) in 38% of patients resistant to vemurafenib after treatment, compare to before treatment and corroborated with in vitro data. The regulation of ID3 expression was related with sensitivity to vemurafenib [105]. In that direction the implication of the ERK/SOX10/FOXD3/ERBB3 axis has been described as an underlying alternative mechanism behind vemurafenib resistance. This work also describes a novel transcriptional regulation mechanism of SOX10 that implicates phosphorylation and sumoylation [106]. The presence of a recently discovered NRAS isoform 2 has been also implicated in the appearance of vemurafenib resistance in cell lines and in tumor samples from melanoma patients. This expression is linked to a reduction of MAPK pathway signaling and to an increase of PI3K pathway signaling. NRAs isoform 2 has been demonstrated to interact with PI3K and BRAF/RAF1 through immunoprecipitation experiments. Partial restoration of sensitivity can be attained by treatment with AKT inhibitors [107]. A novel mechanism that implicates the fusion protein AGAP3-BRAF^V600E^ has been described through a comprehensive genomic profiling of serial biopsies from patients presenting tumor relapse. Interestingly after treatment withdrawal, the fusion protein was not detected in rebiopsies from patients, consistent with fast clonal dynamics in response to treatment. The tumor was still retaining sensitivity to the combination of vemurafenib with MEK inhibitors as the rebiopsies showed after treatment with the two compounds. These data give a rationale for rechallenging with BRAF inhibitors after a certain time in some clinical conditions [108]. Another recently described mechanism involves the loss of stroll antigen STAG2 and STAG3. These proteins have been found decreased in samples from patients that have developed vemurafenib resistance. Loss of STAG2 inhibited the expression of DUSP6, mediating the reactivations of MAPK signaling (through ERK1/2) reinforcing the idea that ERK reactivation as one of the most important mechanisms behind the BRAF inhibitors resistance [109]. Also new mechanisms described behind vemurafenib resistance implicate the hedgehog family of transcriptions factors. Increased levels of GLI1 and GLI2 proteins (independent of canonical Hh pathway) were elevated in cells derived from a melanoma cells with acquired in vitro vemurafenib resistance compared to naive cells, a process that involved the intervention of TGF beta /SMAD signaling. Treatment with the GLI1 and 2 inhibitor Gant61 decrease invasion levels in a 3D skin model with decreased levels of metalloproteinases MMP2 and MMP9 and MITF over expression, and inducing apoptosis. The alternation of vemurafenib and Gant61 prevented the appearance of vemurafenib resistance, pointing to a possible therapeutic strategy in order to prevent melanoma relapse in vemurafenib treated patients [110]. Another work has described Beta-catenin stabilization and nucleus translocations as a key played in approximately half of the melanomas that developed vemurafenib resistance, but through a mechanism that is independent of the canonical Wnt pathway, partly explaining the contradictory results existing about beta-catenin involvement in melanoma. Beta catenin interacts with STAT3, cooperating in the acquisition and maintenance of the resistance [111].

Concomitant expression of a truncated form of BRAF^V600E^ and a point mutated transcriptional repressor BCORL1 is also responsible for the resistant phenotype to vemurafenib in A375 cells. Treatment with the pan-RAF inhibitor sorafenib synergizes with the BRAF inhibitor in order to effectively overcome vemurafenib resistant in these cells [112].

Hypoxia is a driven mechanism implicated in the malignant transformation of multiple cancers. In the case of melanoma, hypoxia is a mechanism that also drives resistance to vemurafenib through HGF/MET induction. Using 3D spheroid cell culture models and two dimensional hypoxic cultures the investigators described the induction of HGF/MET factor as a key player in the acquisition of resistance in hypoxic conditions compare two normoxic. These data were correlated with findings in tissue samples from resistant patients and xenograft models. Inhibition of the MET pathway restored sensitivity to BRAF inhibitors in melanoma cells cultured in hypoxic conditions [113].

miRNA has been also implicated in the appearance of vemurafenib resistance. The work by Díaz-Martinez et al. has revealed that miR-204-5p and miR-211-5p are implicated in this resistance. Using an in vitro generated vemurafenib resistant A375 melanoma cell line as a model, the investigators showed that the expression of miR-204-5p and miR-211-5p was unregulated through RNA stabilization, and the joint over expression stimulated RAS and MAPK expression upon vemurafenib treatment [114].

The introduction of CRISPR technology has resolved some important questions in biology, and resistance to vemurafenib inhibitors could not be an exception. For instance, CRISPR-Cas9 activation screening technology has allowed the identification of 11 lncRNA loci related to the resistance to vemurafenib in melanoma. The transcriptional activation of the locus EMICERI resulted in the activation of four proteins, one of them contributing to vemurafenib resistance [115]. Another recent work using CRISPR-Cas9 technology discovered the CUL3 locus to be implicated in vemurafenib resistance [116].

As important as describing the mechanism that lies behind acquisition of resistant to BRAF inhibitors is finding therapeutic solutions in order to solve this conundrum. To overcome the dual resistance to BRAF and MEK inhibitors, the group of Theodasakis et al. have described that the blockade of p90RSK by BI-D1870 restores sensibility through G0/G1 arrest and induction of apoptosis, offering a new strategy in order to defeat acquired resistance, and also helping to understand the molecular mechanisms behind resistance acquisition [117]. Similar results were obtained by Kosnopfel et al. making even more attractive this approach to alter dual resistance in melanoma [118]. Another mechanism that could be potentially explored in vemurafenib resistant patients is the TGF beta addiction that presents cells that are resistant to BRAF inhibition. This work also demonstrated a paradoxical effect of vemurafenib at low doses in naive cells, inducing proliferation, probably through paradoxical MAPK activation. Inhibition of TGFBR1 blocked SRC phosphorylation and cell growth, and TGFBR1 sensitivity was retained by vemurafenib resistant tumor cells, showing a possible way to circumvent BRAF inhibition resistance in melanoma patients [119]. In this direction, targeting IGFBP1 can be useful in order to overcome vemurafenib resistance [120]. These authors previously reported the over expression of IGF2BP1 in metastatic melanoma and that this expression is implicated in chemotherapeutic resistance [121].

## 6. Combination of Various Anti-Neoplastic Agents in Melanoma Therapy

Vemurafenib and metformin were combined to investigate the effect of the two drugs in the BRAF^V600E^ mutant cell lines. Metformin is a derivative of guanidine and can inhibit mitochondrial ATP production and activate AMP- activated protein kinase (AMPK). The serine/threonine liver kinase B1 (LBK1) is a tumor suppressor gene, which when phosphorylated by ERK and p90, will no longer bind and activate AMPK. In BRAF^V600E^ cell lines there is an uncoupling of LKB1-AMPK complex, which results in cancer cells avoiding apoptosis. The MAPK and AMPK pathways seem to be interlinked and may influence resistance of cancer cells to therapy. Synergistic effects of both drugs were observed in 6 out of 11 BRAF^V600E^ mutant cell lines and 6 out of 7 NRASQ61 mutant cell lines. The reactivation of AMPK by vemurafenib and stimulation of AMPK by metformin might have induced cells to apoptosis due to their interdependency [122].

Vemurafenib has also been shown to improve the antitumor activity mediated by ACT. It was previously postulated that blocking the MAPK pathway with vemurafenib may alter the T lymphocyte function and decrease the immunity of the patient, but that proved to be otherwise [123]. Koya et al. performed an experiment involving a murine model on the melanoma cell line, SM1, that was derived from BRAF^V600E^ transgenic mice. Chicken ovalbumin (OVA) is expressed on SM1-OVA tumors, and the lymphocytes genetically modified with T-cell receptor that recognized OVA were used in the adoptive cell transfer. These cells contained the BRAF^V600E^ mutation and were treated with vemurafenib. The combination increased T lymphocytes function against the tumor with the BRAF^V600E^ mutation. The drug blocked the oncogenic activities in tumor cells to increase self-surface ligands to be presented to the lymphocytes as an immune upregulation. Those lymphocytes that were exposed to the BRAF inhibitor do however have a higher ERK phosphorylation and an increase in immune activation due to the paradoxical activation of the MAPK pathway in WT cells by vemurafenib. Therefore, vemurafenib can be used in combination with ACT to yield positive results on battling with melanoma [124].

## 7. Mechanisms of Melanoma Circumvention of Vemurafenib-Mediated Cytotoxicity

Many drugs have been introduced into the market to combat melanoma. However, many of them fail to decrease cancer proliferation, survival, or growth. As a result, resistance mechanisms develop and cancer cells escape the treatments. Alternative strategies need to be investigated to impede tumor progression and resistance. One of the possible treatments that could be deemed auspicious against battling cancer is the use of posttranslational modifying agents through epigenetic modification. These enzymes are epigenetic modifiers, which cause chromatin remodeling that influence gene expression in malignant cancer cells without changing the DNA sequence in an individual. Some of these modifications include demethylation, histone deacetylation inhibition, and histone phosphorylation. Proteasome inhibitors might also play a role in the battle against cancer cell proliferation. All of the agents facilitate apoptosis and cell cycle arrest of the tumor cells [125,126,127]. Histone methyltransferases (HMT) are associated with chromatin remodeling and transcriptional silencing of genes at lysine residues. Histone demethylases remove methyl groups from histones, which counter the effect of HMT [128]. Using the effect that these agents induce could potentially damage the chromatin structure of melanoma cancer cells, which will make the cells more sensitive to apoptosis and vemurafenib. Histone phosphorylation occurs at serine and threonine residues, which alters the charge of the amino acid and leads to chromatin remodeling. ERK 1 and ERK2 of the MAPK pathway are involved in phosphorylation of serine 10 within H3, which leads to a condensed chromatin structure and abnormal expression of genes [129,130]. Inhibiting the phosphorylation will deactivate the MAPK pathway, which is constitutively activated with a BRAF^V600E^ mutation. Interfering with the kinases will counteract the resistance of the cells.

When DNA methyltransferases (DNMTs) add a methyl group to 5′ carbon of cytosine catalyzing a modification of cytosine, DNA methylation takes place [131]. Hypermethylated CpG islands silence transcription of tumor suppressing genes, which may cause tumor cell proliferation [132]. DNA methyltransferase inhibitor 2′-deoxy-5-azacytidine, known as decitabine, inhibits antitumor activity by demethylation. It forms a DNA-protein complex and inhibits DNMT activity and the targets are no longer methylated [133,134,135]. In melanoma patients who were treated in with decitabine, 22% attained responses resulting in a stable disease [136]. Demethylating agents stimulate reactivation of tumor suppressor genes, which results in inhibition of tumor growth. Tumor suppressor Apaf-1 mediates p53, and when hypermethylated it is silenced. Once silenced, the protein no longer maintains its purpose in apoptosis. Decitabine restores the expression and renders the melanoma cells to apoptosis [137]. Since DNA methyltransferase inhibitors target proliferating cells, they could be used against constitutive cancer cells such as those with aberrant MAPK pathway activation. Recently, a minor subpopulation of melanoma cells expressing a histone modifier JARID1B was found. This molecule is a histone demethylase that might contribute to a promising drug treatment [138].

Other evidence suggests that hypomethylation could also be promoted by BRAF^V600E^ in melanoma tumors. Some genes become hypermethylated once BRAF is knocked down [139]. Hypomethylation can cause silent regions to become activated, which might yield an overexpression of genes and cause cells to proliferate uncontrollably like in cancer [140]. Exploitation of DNA and histone hyper- and hypomethylation inhibitor testing in patients with BRAF^V600E^ mutation could be of a benefit by decreasing resistance to vemurafenib with epigenetic alterations.

Chromatin that has low levels of acetylation on the lysine residues of NH2 terminal tails is transcriptionally silent. Acetylation of chromatin is controlled by histone acetyltransferases (HATs) and histone deacytylases (HDACs) [141,142]. Aberrant activity of HDACs may lead to tumor cell growth and proliferation. Some non-histone protein targets of HDACs are transcription factors, regulators, signal transduction mediators and DNA repair enzymes. HDACs could overexpress repressive transcription factors [143]. HDAC inhibitors produce cell cycle arrest and induce apoptosis that silence genes by acetylation of histones. High concentrations of HDAC inhibitors induce G1 and G2/M cycle arrest and decrease cyclin D1 and CDK activity. The decreased CDK activity is due to the upregulation of the cyclin inhibitor p21 that is induced by the HDAC inhibitors [144,145,146]. Cyclin D1 contributes to vemurafenib resistance as stated previously. Lai et al. also demonstrated that cell lines that are resistant to vemurafenib undergo apoptosis induced by PLX4720 in combination with HDAC inhibitors [147]. HDAC inhibitor, MS-275, was tested on two melanoma patients in Phase I, and the drug seemed to induce long lasting, near complete remission [148].

HDAC inhibitor sodium butyrate radiosensitize melanoma cell lines to ionizing radiation and suppress DNA repair activity, and inhibition of HDACs by sodium butyrate leads to increased p53 acetylation and upregulation of Bax, a proapoptotic protein [149,150]. Using other HDAC inhibitors such as suberoylanilide hydroxamid acid (SAHA), valptotic acid (VPA), and trichostatin A (TSA), might also induce patient tumor remission and increase the chances of overall survival. In a study conducted by Facchetti in 2004, TSA and SAHA inhibited proliferation of melanoma cells, with longer bioavailability than short chain fatty acids, and the activity of VPA had a 50% decrease of proliferation of the tumor cells [151].

Proteasomes serve as a major pathway in protein degradation, and ubiquitinated proteins are the major targets in the proteasome complex [152]. Eventually the proteins are degraded. Bortezomib (PS-341) is a proteasome inhibitor that was associated with growth-inhibitory effects that have pleiotropic action. Proteasome inhibition enhances cell death induced by chemotherapy and radiotherapy, even though indirectly [125,153]. Bortezomib also sensitizes melanoma cells towards ACT, which activates apoptotic machinery. Vemurafenib treated cells might acquire defiance also because of resistance to cytolytic T lymphocytes [154]. When bortezomib was combined with temozolomide, tumor growth was reduced [155]. If used along with vemurafenib, the synergistic effect might induce the melanoma cells to apoptosis due to CTL sensitivity.

In order for RAS proteins to be activated they need to be localized to the cell membrane. This requires posttranslational modifications of farnesylations, geranylgeranylation, and methylation of RAS. The inhibitor of farnesyltranferases that target RAS is tipifarnib [156,157]. Inhibiting these posttranslational modifications will decrease the chances of RAS localization and as a default inhibit the MAPK pathway, which in combination with other posttranslational modifying agents might render the cells to apoptosis. Chromatin remodeling along with the combination of proteasome inhibitors might play a role in resynthesizing cells to BRAF inhibitors, allowing a different therapeutic approach towards battling melanoma.

From all clinical trials conducted yearly against melanoma, it is unlikely that one solitary regimen and counteracting one therapeutic target will cure patients. Discovery of new treatment options represent a severe necessity of novel research along with personalized therapy. Potential drug therapies that overcome or prevent resistance to inhibitors must be further investigated and clinical trials should proceed. The low response rates of treatments should serve as encouragement to develop clinical research for future direction and fruitful outcomes. Identification of various origins and mutations in tumors will elucidate alternative remedies. Synergistic therapy against cancer will target multiple pathways of activation and prevent initial causes, which could cause toxicity to the patient yet might be the most auspicious route for progression free survival for those suffering from melanoma.
